# Vaginal Microbial Environment Skews Macrophage Polarization and Contributes to Cervical Cancer Development

**DOI:** 10.1155/2022/3525735

**Published:** 2022-08-09

**Authors:** Guannan Zhou, Fangyue Zhou, Yuanyuan Gu, Menglei Zhang, Ganrong Zhang, Fang Shen, Keqin Hua, Jingxin Ding

**Affiliations:** ^1^Department of Gynecology, The Obstetrics and Gynecology Hospital of Fudan University, 419 Fang-Xie Road, Shanghai 200011, China; ^2^Shanghai Key Laboratory of Female Reproductive Endocrine Related Diseases, Shanghai 200011, China; ^3^The International Peace Maternity and Child Health Hospital, School of Medicine, Shanghai Jiao Tong University, Shanghai, China

## Abstract

As a common female reproductive system malignancy, cervical cancer (CC) disturbs numerous women's health. This study demonstrates the role of the vaginal microbial environment (*Peptostreptococcus anaerobius*) in cervical cancer. Functional assays, including cell proliferation assay, tube formation assay, and immunofluorescence staining, revealed the effect of *Peptostreptococcus anaerobius*-treated macrophages on cell proliferation and the angiogenesis process. The tube formation assay disclosed the function of *Peptostreptococcus anaerobius*-treated macrophages on angiogenesis. In vivo assays were also established to explore the impact of *Peptostreptococcus anaerobius*-treated macrophages on tumor migration. The results revealed that *Peptostreptococcus anaerobius*-induced macrophages boosted cervical cancer migration and angiogenesis both in vitro and in vivo. Then, this study unveiled that *Peptostreptococcus anaerobius*-induced macrophage secreted VEGF to stimulate the angiogenesis in cervical cancer. As a whole, *Peptostreptococcus anaerobius*-induced macrophage facilitates cervical cancer development through modulation of VEGF expression.

## 1. Introduction

It was widely acknowledged that the healthy physiological process depends on the healthy and balanced vaginal microbial ecosystem [1]. As the development of sequencing technology, the role of the microbial environment in the body is widely demonstrated [2, 3]. Many studies have reported that bacterial vaginosis is associated with many reproductive issues [4]. For example, the adverse obstetric outcomes are related to pelvic inflammatory diseases [5] and PCOS [4, 6]. Several studies [7–9] have reported that the variation of the vaginal microbe composition influences the infection status of *Human papillomavirus* (HPV). Our previous study indicated that the composition changes are correlated with the cervical precancer lesions and HPV infections [10]. However, whether the vaginal microbes determine the immune response in the tumor microenvironment and further affect the tumor progression including migration and angiogenesis is unclear.

Cervical cancer is one of the main malignancies in the female reproductive system [11], while the persistent high-risk HPV infection [12] is the main cause of most cervical precancer and cervical cancers. The increasing evidence indicates that the vaginal microbial environment is associated with the progression of female diseases, including infertility, PCOS, inflammation [13], and cancers [14, 15]. So, what is the relationship between vaginal microbe and cervical cancer? And how the vaginal microbial environment exerts the functions in the progression of cervical cancer is still unclear.

In this study, we figured out the differential expressed vaginal microbes in cervical cancer women and then demonstrated the effect of the *Peptostreptococcus anaerobius* on the macrophage polarization, further regulating the angiogenesis in vitro and migration in vivo via secreting VEGF.

## 2. Methods and Materials

### 2.1. Cell Culture

Cervical cancer SiHa cells and SiHa-Luciferase were gifts from the laboratory of the Obstetrics and Gynecology Hospital of Fudan University and maintained in RPMI-1640 medium (E600028, Sangon Biotech, Songjiang, Shanghai, China), supplemented with 10% FBS (Gibco, Guangzhou, Guangdong, China), 1% penicillin (Sangon Biotech, Songjiang, Shanghai, China), and 1% streptomycin in a humidified incubator with 5% CO_2_ at 37°C. HUVEC cells were maintained in DMEM medium supplemented with 10% FBS as well as 1% penicillin & streptomycin in a humidified incubator with 5% CO_2_ at 37°C.

### 2.2. Ethics Approval

The study was approved by the institutional review board of the Obstetrics and Gynecology Hospital of Fudan University. All the study processes were implemented based on the Declaration of Helsinki. Also, the study obtained oral informed consents and written informed consents. All animal experiments were housed and maintained in a standard environment, and the protocol was reviewed and approved by the animal care and research committee of Fudan University.

### 2.3. Tissue Samples

The ethical approval of this study was acquired from the ethics committee of the hospital, and written informed consents were provided by all participants. A total of 20 of cervical squamous cell carcinoma samples (10 cervical squamous cell carcinoma samples with lymphatic metastasis positive and 10 cervical squamous cell carcinoma samples with lymphatic metastasis negative as control) were acquired from patients who receive surgery and were collected for this study between January 2019 and March 2020. The exclusion criteria were as follows: (i) history of other drug treatment before surgery, (ii) history of cervical conization surgery, (iii) other pathological types of cervical cancer carcinoma, (iv) other female reproductive system malignancies, and (v) non-postmenopausal. Tissue samples were all snap frozen in liquid nitrogen and preserved at −80°C, for further measurement.

### 2.4. Cell Viability Assay

The cell viability of HUVEC cells was evaluated by CCK8 assays. Totally, 10^4^ cells/well were seeded into the 96-well plate with 100 *μ*L DMEM for 12 h and cells were added with *Peptostreptococcus anaerobius*-treated macrophage medium, blank bacterium medium-treated macrophage medium, and PBS for 48 h. Then, the CCK-8 kit was added into the medium in wells for 1 hour at 37°C. The cell viability was evaluated by the OD450 value of each well.

### 2.5. Tube-Formation Assay

Matrigel was evenly distributed to every well in a 96-well plate for 30 min at 37°C. HUVEC cells at early passage were prepared and added into each well for 3 hours and captured under the microscope. Tube formation was assessed under the microscope.

### 2.6. ELISA Assay

After being treated with diverse medium, the cell supernatant sample collection was conducted. Briefly, the detection of the serum levels of VEGF was conducted by the ELISA kits (Yanhui, Shanghai, China) (^∗∗∗^*p* < 0.001).

### 2.7. Immunofluorescence Assay

HUVEC cells were seeded on a slide in the 24-well plate. Immunofluorescence images of VEGFR2 expression on HUVECs were captured, after treatment with indicated media (Peptostreptococcus anaerobius treated macrophage media) stimulation for 72h. Cells were fixed with 10% paraformaldehyde, the VEGFR were stained by FITC-VEGFR antibody, and nuclei were stained with 4,6-diamidino-2-phenylindole (DAPI) (Millipore) for 10 min at 4°C. Images were taken using a Leica fluorescent microscope and a TCS SP5 confocal laser scanning microscope (Leica Microsystems, Wetzlar, GER).

### 2.8. Animal Assay

Mice were divided into three groups: (i) *Peptostreptococcus anaerobius*-treated macrophage group, (ii) blank medium-treated macrophage group, and (iii) PBS group. Female athymic nude mice (4 weeks old) were purchased from JieSiJie Laboratory Animal Co. Ltd., Shanghai, China. For bioluminescence evaluation in the mouse model, we cultured SiHa-Luciferase stable transfected cells. SiHa-Luciferase cells (2 × 10^6^/mL) were seeded into nude mice via intraperitoneal injection. Medium gathered from *Peptostreptococcus anaerobius*-treated macrophages, medium from blank medium-treated macrophages, and PBS were injected into mice intraperitoneally for two times a week and totally 3 weeks. Mice were analyzed by a live imaging upon animals.

### 2.9. Data Analysis

In this study, we conducted the correlation analysis not only for the gene expression evaluation but also for the survival prognosis evaluation in cervical carcinoma, which is based on the TCGA database (https://tcga-data.nci.nih.gov). Furthermore, we conducted analysis (including the expression analysis and the survival analysis) based on the GEPIA2 website (http://gepia2.cancer-pku.cn/). All the experiments were repeated in triplicate, and experimental results were expressed as the means ± standard deviation (S.D.). We use Student's *t*-test or one-way ANOVA to determine statistical probabilities, with a *p* value below 0.05 as a significant level. And we used the SPSS 25.0 software (IBM Corp., Armonk, NY) to analyze the data. Gene linear correlation was analyzed by Pearson correlation analysis.

## 3. Results

### 3.1. Peptostreptococcus anaerobius Is Upregulated Expressed in Cervical Cancer Cervicovaginal Lavage Fluid and Tissues

**Figure 1:**
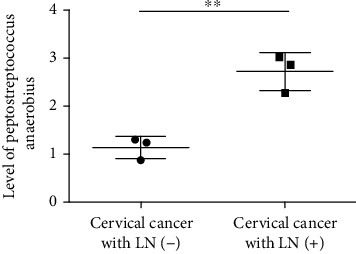
The level of *Peptostreptococcus anaerobius* in cervical cancer.

At first, the expression of *Peptostreptococcus anaerobius* in 20 participants (including 10 women (cervical squamous cell carcinoma lymphatic metastasis positive) with cervicovaginal lavage fluid and 10 women (cervical squamous cell carcinoma lymphatic metastasis negative, as control) with cervicovaginal lavage fluid) was examined. As depicted in Figure 1, it was revealed that *Peptostreptococcus anaerobius* was significantly highly expressed in cervicovaginal lavage fluid of cervical cancer women, when compared with healthy women. As depicted in our previous study, the level of *Peptostreptococcus anaerobius* in cervical cancer lesion was higher than that of nontumor women.

### 3.2. The Relationship between the M2 Phenotype and Peptostreptococcus anaerobius in Cervical Cancer

**Figure 2:**
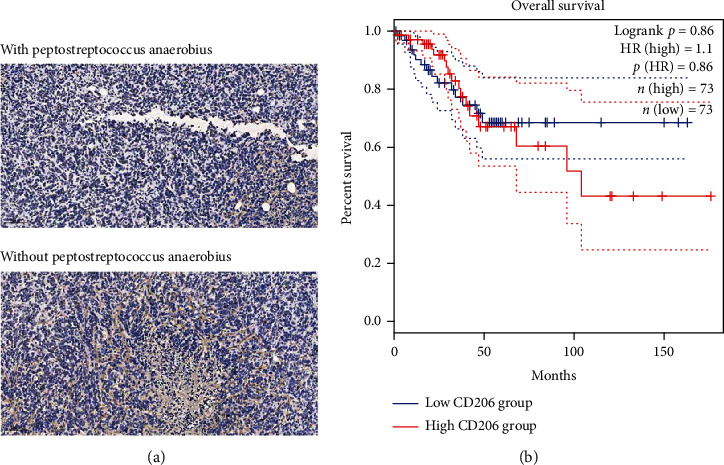
The expression of CD206 in cervical cancer. (a) The IHC staining of cervical carcinoma tissues upon the CD206 in cervical cancer tissues from women with *Peptostreptococcus anaerobius* (down) and in cervical cancer tissues from women without *Peptostreptococcus anaerobius* (upper). (b) The analysis of CD206 expression via TCGA set base: the increased expression level of CD206 was relevant to the lower survival rate and inferior prognosis (blue curves); the decreased expression level of CD206 was relevant to the higher survival rate and superior prognosis (red curves).

To investigate the correlation between infiltration of tumor-associated macrophages and *Peptostreptococcus anaerobius* in cervical cancer, we performed IHC staining to detect the M2 macrophage marker CD206 in cervical cancer tissues derived from women with *Peptostreptococcus anaerobius* and cervical cancer tissues derived from women without *Peptostreptococcus anaerobius*. As shown in Figure 2(a), we observed elevated infiltration of CD206-positive M2 macrophage infiltration in cervical cancer tissues from women with *Peptostreptococcus anaerobius*, when compared with women without *Peptostreptococcus anaerobius*. In addition, overall survival analysis showed that the overall survival rate of the higher expression of the CD206-positive group in cervical cancer was better than that of lower expression of the CD206-positive group. Also, we conducted the analysis about the survival prognosis in cervical cancer patients. As depicted in Figure 2(b), the increased expression level of CD206 was relevant to the lower survival rate and inferior prognosis, while the decreased expression level of CD206 was relevant to the higher survival rate and superior prognosis.

### 3.3. The Effect of *Peptostreptococcus anaerobius* to Induce the Macrophage into the M2 Phenotype

**Figure 3:**
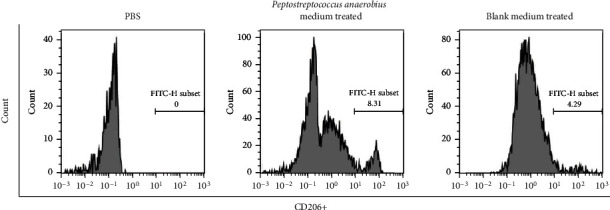
The effect of *Peptostreptococcus anaerobius* upon the polarization of macrophages: the expression of CD206 (M2 macrophage markers) in THP-1 cells when treated with PBS or *Peptostreptococcus anaerobius* medium or blank medium.

To determine whether *Peptostreptococcus anaerobius* induced M2 polarization of macrophages, we first selected THP-1 cells and induced THP-1 cells into the M0 macrophage by PMA and then treated M0 macrophages with collected *Peptostreptococcus anaerobius* medium. As depicted in Figure 3, the results showed that the expression of M2 markers (CD206) in PMA-treated THP-1 cell-administered blank medium or *Peptostreptococcus anaerobius* medium was apparently lower or higher, respectively. Taken together, the abovementioned results confirm that *Peptostreptococcus anaerobius* can induce M2 polarization of macrophages.

### 3.4. The Effect of *Peptostreptococcus anaerobius*-Treated Macrophage to Induce the Angiogenesis

**Figure 4:**
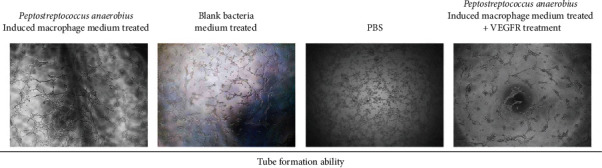
The effect of *Peptostreptococcus anaerobius* upon tube formation for detecting angiogenesis ability. The tube formation ability of HUVECs after being treated with medium from *Peptostreptococcus anaerobius*-induced macrophages, blank bacteria medium-induced macrophages, PBS, and medium from *Peptostreptococcus anaerobius*-induced macrophages plus VEGFR.

In order to analyze the effect of *Peptostreptococcus anaerobius*-treated macrophage on regulating the angiogenesis process, we conducted the viability assay and tube formation assay by using the HUVEC cells. As shown in Figure 4, the tube formation assay revealed that the induction of *Peptostreptococcus anaerobius*-treated macrophages on tube formation efficiency was higher than the blank medium-treated macrophages and PBS-treated macrophages.

### 3.5. The *Peptostreptococcus anaerobius*-Treated Macrophages Secrete VEGF to Induce Angiogenesis

**Figure 5:**
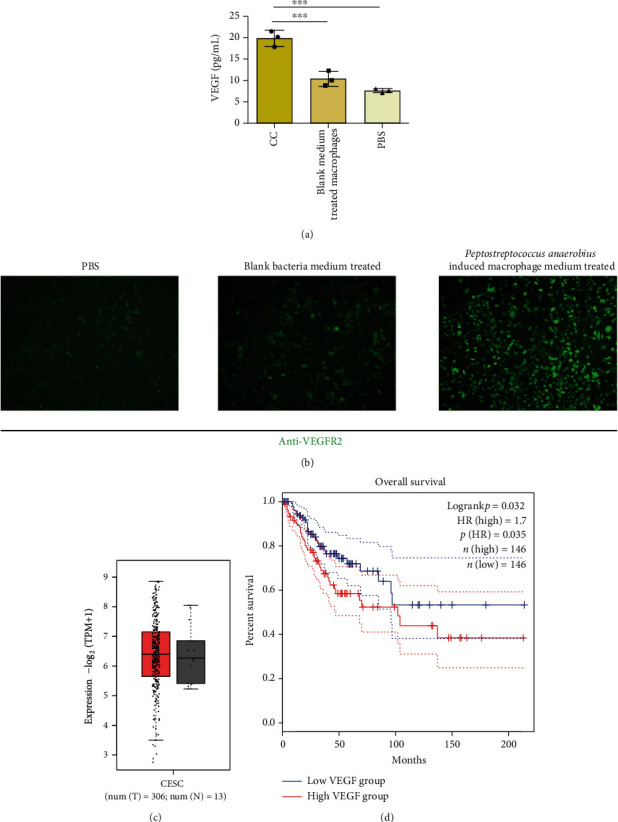
The expression level of VEGF from macrophages treated with *Peptostreptococcus anaerobius*. (a) The expression level of VEGF from macrophage medium after being treated with medium from *Peptostreptococcus anaerobius*-induced macrophages, blank bacteria medium-induced macrophages, and PBS. (b) The VEGFR expression on HUVECs after being treated with medium from *Peptostreptococcus anaerobius*-induced macrophages, blank bacteria medium-induced macrophages, and PBS. The expression analysis (c) and overall survival analysis (d) of VEGF in cervical cancer via TCGA set base.

Since *Peptostreptococcus anaerobius*-treated macrophages expressed markers of the M2 phenotype, and M2 macrophages were reported to secret cytokines and growth factors, such as VEGF and PDGF. In order to explore how the *Peptostreptococcus anaerobius*-treated macrophages upregulate the ability of angiogenesis, we conducted the ELISA assay to evaluate the VEGF expression of the *Peptostreptococcus anaerobius*-treated macrophages, blank bacteria medium-treated macrophage medium, and control groups. As depicted in Figure 5(a), the results revealed that VEGF expression was significantly upregulated in *Peptostreptococcus anaerobius*-treated macrophage medium than in the blank bacterium-treated macrophage medium or the control group. While we added the VEGF receptor protein into the HUVECs with medium from *Peptostreptococcus anaerobius*-treated macrophages, the induced angiogenesis process was inhibited. What is more, in Figure 5(b), the *Peptostreptococcus anaerobius*-treated macrophage medium could increase the expression level of VEGFR2 in HUVEC cells. In addition, as depicted in Figures 5(c) and 5(d), the cervical cancer tissue expressed a higher level of VEGF and the increased expression level of VEGF was relevant to the lower survival rate and inferior prognosis.

### 3.6. The Effect of *Peptostreptococcus anaerobius*-Treated Macrophages on Inducing the Migration of Cervical Cancer in the Animal Model

**Figure 6:**
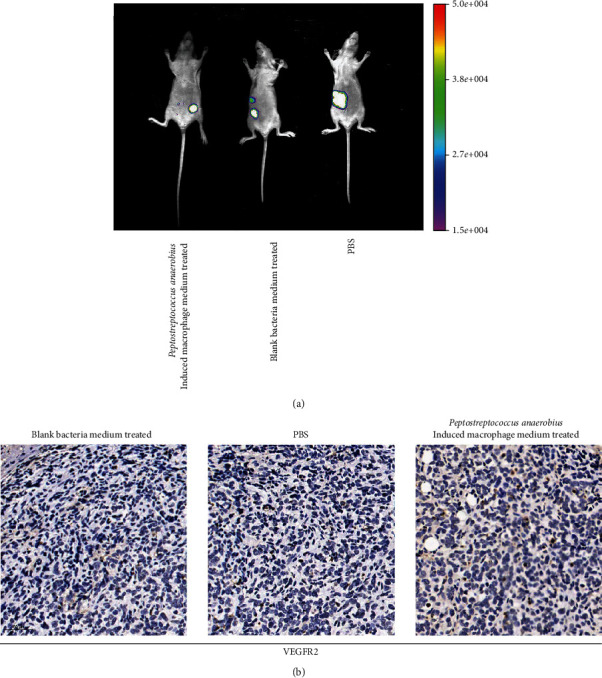
The effect of VEGF from macrophages treated with *Peptostreptococcus anaerobius* on the migration in cervical cancer in vivo. (a) Bioluminescent images were visualized to evaluate the metastasis of cervical cancer cells after being treated with *Peptostreptococcus anaerobius*-induced macrophage-conditioned medium, blank bacteria medium-induced macrophage-conditioned medium, and PBS in the mouse model. Luminescence of cervical cancer cells was evaluated by an in vivo imaging system, in which the size of the luminescence area represents the metastasis ability. (b) The IHC staining for detecting the VEGFR expression in tumor after being treated with blank bacteria medium-induced macrophage-conditioned medium, PBS, and *Peptostreptococcus anaerobius*-induced macrophage-conditioned medium.

In in vivo assay, we used the SiHa-luciferase cancer cells for evaluating the effect of *Peptostreptococcus anaerobius*-treated macrophages on the cervical cancer. We divided animals into three groups: *Peptostreptococcus anaerobius*-treated macrophage group, blank medium-treated macrophage group, and PBS-treated group. Luminescence of cervical cancer cells was evaluated by an in vivo imaging system to demonstrate the cancer metastasis in vivo. As demonstrated in Figure 6(a), *Peptostreptococcus anaerobius*-treated macrophages enhanced the migration of cervical cancer in vivo, when compared with blank medium-treated macrophages or PBS. In addition, the VEGFR2 expression level in tumor resected from the *Peptostreptococcus anaerobius*-treated macrophage group is higher than that from the blank medium-treated macrophage groups or PBS group (Figure 6(b)).

## 4. Discussion

Cervical cancer is the one of the main female reproductive system malignancies, which disturbs the health of women around the world. Although, with the development of screening strategy [16] and the popularization of HPV vaccine [17], the morbidity and mortality decreased in developed countries [18]. However, cervical carcinomas still result in numerous deaths in developing countries. Nowadays, increasing evidence indicate that vaginal microbes are associated to the disease process [19], including inflammations [20], infertility [21], and cancers [22]. In the current study, we demonstrated that *Peptostreptococcus anaerobius*, a kind of vaginal microbe, is expressed rarely in healthy women, promotes the macrophage polarization in the tumor microenvironment, and further induces the angiogenesis process in vitro and migration in vivo. Mechanistically, the *Peptostreptococcus anaerobius*-induced macrophage expressed the M2 phenotype and the *Peptostreptococcus anaerobius*-induced macrophage could secret VEGF to induce the angiogenesis process. VEGF is a specific angiogenesis factor and could stimulate endothelial tube formation to generate new vessels. A previous study [23] revealed that VEGF could induce the angiogenic process upon endothelial cells. These findings indicate that the vaginal microbes exert certain functions in the development of cervical cancer.

It was of significance to demonstrate the effect of the vaginal microbial environment in the physiological process and pathological process in the female reproductive system. It was reported [24, 25] that there are significant differences in vaginal microbiome between cancer microenvironment and noncancer microenvironment women, but less studies reported the mechanisms among these associations. In this study, we explore the effect of *Peptostreptococcus anaerobius* on macrophage polarization and further angiogenesis and also explore the certain mechanisms of these effects.

To our knowledge, this is the first study to demonstrate the effect of *Peptostreptococcus anaerobius* on macrophage polarization in the tumor microenvironment in cervical cancer. This study is based on our previous analysis by means of comparing the expression differences among the different status of cervical precancer lesions.

There are also many limitations to this current study. Firstly, more relative samples of cervical cancer tissues are needed to verify the relations between the macrophage infiltration and vaginal microbe; also, more samples of cervicovaginal lavage fluid are needed in the future study. Secondly, further more studies shoube be conducted to figure out if there are other vaginal microbials could play roles in regulating the vaginal microenvironment in cervical cancer. What is more, further experiments should explore whether the expression of specific vaginal microbes indicates the specific prognosis in cervical cancer.

## 5. Conclusions

We demonstrated that vaginal microbial *Peptostreptococcus anaerobius* could contribute to cervical cancer progression via inducing M2 macrophage polarization. In addition, we revealed that *Peptostreptococcus anaerobius*-induced macrophage could promote the angiogenesis in vitro and in vivo. Furthermore, we found that *Peptostreptococcus anaerobius*-induced macrophage could promote the angiogenesis via secreting VEGF. Thus, our study elucidated a novel molecular mechanism promoting cervical cancer underlying the interaction between vaginal microbes, macrophages, and tumor cells. These results will contribute to the new insights of the development of cervical cancer. Also, the results would contribute to the effective preventive and therapeutic strategies for cervical cancer. More importantly, high *Peptostreptococcus anaerobius* in cervicovaginal lavage fluid was correlated with cervical cancer, suggesting that it may be a promising biomarker for liquid biopsy and predicting the risk of cervical cancer in the future. In addition, targeting the vaginal microbial-mediated crosstalk between tumor cells and macrophages may provide novel strategies for the treatment of cervical cancer.

## Data Availability

Data are available form the corresponding authors once needed.
